# Effects of IPR by mechanical oscillating strips system on biological structures: a quantitative and qualitative evaluation

**DOI:** 10.1186/s40510-023-00460-9

**Published:** 2023-03-13

**Authors:** Francesca Gazzani, Denise Bellisario, Laura Fazi, Alessia Balboni, Silvia Licoccia, Chiara Pavoni, Paola Cozza, Roberta Lione

**Affiliations:** 1 Department of Systems Medicine, University of Rome “Tor Vergata”, Viale Oxford 81, 00133 Rome, Italy; 2 Department of Industrial Engineering, University of Rome ‘Tor Vergata’, Rome, Italy; 3 Department of Science and Chemical Technology, NAST Centre, University of Rome ‘Tor Vergata’, Rome, Italy; 4 Department of Dentistry, UNSBC, Tirana, Albania; 5 Department of Faculty of Medicine and Surgery, UniCamillus International Medical University, Rome, Italy

**Keywords:** Interproximal enamel reduction, Mechanical oscillating strips, SEM analysis

## Abstract

**Background:**

To evaluate by means of profilometric analysis and scanning electronic microscope (SEM) the effects on enamel surfaces of oscillating mechanical systems for interproximal enamel reduction (IPR). Fifteen complete (Group 1) oscillating IPR sequence and 15 single metallic strips (Group 2) for active IPR phase of 0.2 mm were selected and tested on 30 freshly extracted teeth by means of tribological tests with alternative dry-sliding motion (Linear Reciprocating Tribometer, C.S.M. Instruments, Peseaux, Switzerland). Enamel surface roughness and waviness measurements were assessed by contact probe surface profiler (TalySurf CLI 2000; Taylor Hobson, Leicester, UK) and a TayMap software for the 3D analysis. Statistical analysis was performed with independent samples *t*-test. Significance was established at the *P* < .05 level. SEM analysis of enamel surfaces was conducted with a FEI Quanta 200 (Hillsboro, USA) in high vacuum at 30.00 kV. Images were acquired at 30X, 100X, and 300X of magnification.

**Results:**

Teeth undergone Group 1 showed lower values of surface roughness (Ra − 0.34 µm, Rt − 1.55 µm) and significant increase of waviness parameters (Wa 0.25 µm, Wt 4.02 µm) when compared with those treated with Group 2. SEM evaluation showed smoothers and more regular surfaces when IPR was performed by complete IPR sequence. Single metallic strip determined more irregular surfaces characterized by extended grooves, alternated with enamel ridges and irregular fragments.

**Conclusion:**

The adoption of a standardized oscillating IPR sequence determines more regular and harmonious enamel surfaces at the end of the procedure. An adequate polishing after IPR plays a crucial role to guarantee a good long-term prognosis and a good respect of biological structures.

## Background

Nowadays interproximal reduction (IPR) represents one of the main space-gaining orthodontic procedures in several clinical cases [1, 2], especially in clear aligner treatment [3]. Combined with proclination and transversal expansion, it is a challenging alternative to dental extraction for the resolution of mild or moderate crowding [1]. In these cases, the quantity of enamel removed should be calculated considering the space needed. Other clinical indications include Bolton tooth-size discrepancies, morphologic anomalies, and reduction of interdental gingival papilla retraction [1, 4–7]. Several IPR systems have been introduced over the years [8, 9]. Among all, mechanical oscillating abrasive strips have gained in popularity for their accuracy, efficiency, reduced chairside time, and minimally invasive effects on enamel surfaces [8, 10]. Recently, Gazzani et al. [8] compared mechanical oscillating diamond strips with manual ones. Higher efficiency in terms of enamel reduction and more regular enamel surfaces were observed with mechanical oscillating diamond strips when compared with the manual system. Moreover, it has been widely demonstrated [9, 11, 12] that polishing phase after IPR procedures defines smoother enamel surfaces. A clinically relevant aspect to consider is the necessity of a standardized clinical protocol to follow to not affect surface morphology and to quantify the amount of enamel removed. Accuracy and safety of IPR play a crucial role in the treatment since it ensures the predictability of clinical results and the integrity of the treated surfaces [2, 6]. Regardless the IPR methods, the clinical sequence to follow should consist of some standardized steps [9]: opening phase for the access to the interproximal areas (1); interproximal enamel removal (2); check of enamel removed (3); finishing and polishing phases (4). As a matter of fact, mechanical oscillating systems consist of sequential use of different strips with gradually increasing abrasive properties and some dedicated to polishing phases. The use of a standardized sequence should satisfy the need to exactly quantify the enamel removed and, not least, to preserve the enamel surface from the risk of residual roughness and irregularities. In regard to this issue, the aim of the present study was to evaluate the effects of a mechanical oscillating IPR system on enamel surfaces by means of the tribological test and scanning electronic microscope (SEM). The clinical sequence effects were compared with those of a single oscillating abrasive strip in order to validate the importance to respect the clinical IPR phases required by the protocol.

## Methods

**Fig. 1:**
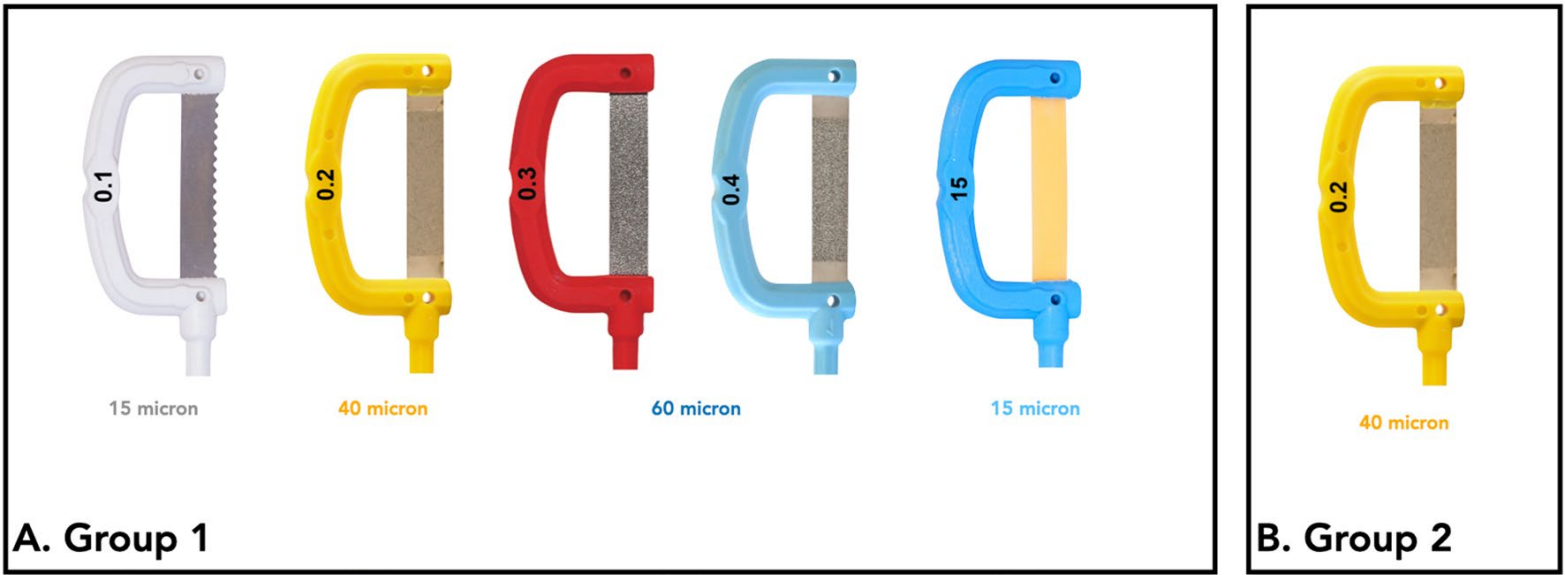
Experimental analysis. **A** Group 1. Complete oscillating IPR sequence including one opener (0.1 mm), three metallic strips for active IPR phase (0.2 and 0.3 mm, 0.4 mm), and one resin strip for polishing phase (0.15 mm). **B** Group 2. Single metallic strip for active IPR phase of 0.2 mm

Fifteen complete oscillating IPR sequences (Group 1; DentaSonic, Cham, Switzerland) including one opener (0.1 mm), threev metallic strips for active IPR phase (0.2 and 0.3 mm, 0.4 mm), and one resin strip for polishing phase (0.15 mm) were collected and tested (Fig. 1A). Fifteen single 0.2 mm metallic strips for active IPR phase (Group 2; Dentasonic, Cham, Switzerland) were selected to be compared with the IPR sequence (Fig. 1B). Thirty teeth were collected and obtained over the years from patients who had an extraction therapy at the Department of Orthodontics, University of Rome “Tor Vergata.” Informed consent agreement was signed by all patients for orthodontic treatment and to allow their teeth to be used for research purposes. Extracted teeth were thoroughly cleaned of debris and soft tissue, then conserved and fixed in 4% glutaraldehyde in 0.2-M sodium cacodylate buffer solution at 48 °C. Each tooth was blocked by acrylic resin in a rectangular pot, designed and manufactured by a 3D printer. The resin block was then positioned in a metallic clamp support to be underwent tribological test.

### Tribological tests and wear evaluation

**Fig. 2:**
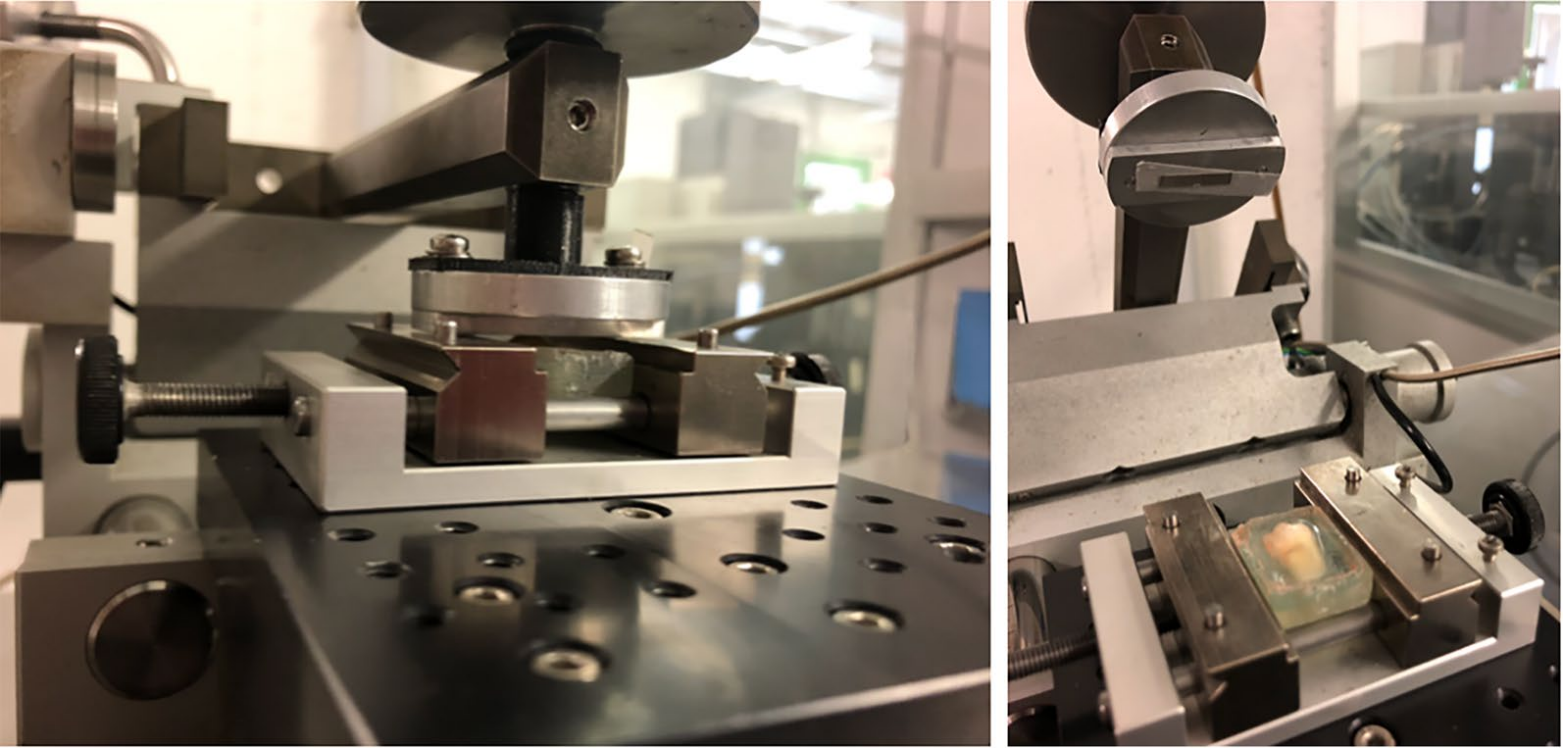
Experimental analysis. Mechanical oscillating strip adapted on the Instron Universal Testing Machine and tooth fixed in the resin support

Tribological tests with alternative dry-sliding motion were performed on samples for both Group 1 and Group 2 by a standard tribometer (Linear Reciprocating Tribometer, C.S.M. Instruments, Peseaux, Switzerland) (Fig. 2). Each selected abrasive strip moved against stationary, freshly extracted mandibular first premolars fixed in resin blocks, at a 1-N load (frequency, 10 Hertz; stroke, 10.4 mm; 300 laps). The sliding time was set at 30 s for each strip of the sequence simulating clinical use conditions. The comparing test with the use of the single 0.2 metallic strip consisted of 5 steps of 30 s each of dry-sliding motion. The testing time lapse was set considering the sliding motion of the metallic strips use during oscillating IPR sequence. Teeth wear was assessed by a contact probe surface profiler (TalySurf CLI 2000; Taylor Hobson, Leicester, UK). The profilometer was used to rebuild the wear patterns using a 5-μm lateral resolution. The profile of each tested tooth was recorded and the following surface roughness and waviness measurements were evaluated with a 0.8 mm Gaussian cutoff filter: arithmetic mean roughness value (Ra, µm), mean peak width (RSm, µm), total height of the roughness profile (Rt, µm), arithmetic mean waviness value (Wa, µm), total height of the waviness profile (Wt, µm). Analysis was performed comparing surface roughness and waviness between surfaces undergone the Group 1 and Group 2. Independent sample *t*-test was used for the statistical analysis of the results. Significance was established at the *P* < 0.05 level. The maximum and mean depth, the area, and the volume involved by the action of the counterpart on the surface of the samples were evaluated by using a TayMap software to calculate and qualitatively analyze the 3D wear patterns.

### Evaluation of effects on enamel surface

At the end of the tribological tests, enamel surface condition was qualitatively evaluated before and after IPR with SEM analysis with a FEI Quanta 200 (Hillsboro, USA) in high vacuum at 30.00 kV at 30X, 100X, and 300X of magnification. A modified version of a scoring scale previously used by Nucci et al. [8, 13] was used to describe enamel surface, and the integrity level of the enamel surface was evaluated as follows:

*Score 0* Enamel surface free of scratches and grooves;

*Score 1* Scratches and grooves not very accentuated and covering a portion of the surface;

*Score 2* Deep furrows with evident rounded edges over the entire surface, without debris;

*Score 3* Evident and deep-edged furrows visible on the whole surface and presence of debris on the enamel.

All measurements were performed by the same researcher (DB). The intra-examiner repeatability of the researcher was analyzed on 15 teeth and it was found to be high (Pearson correlation coefficient: 0.895, *p* < 0.001).

## Results

**Fig. 3:**
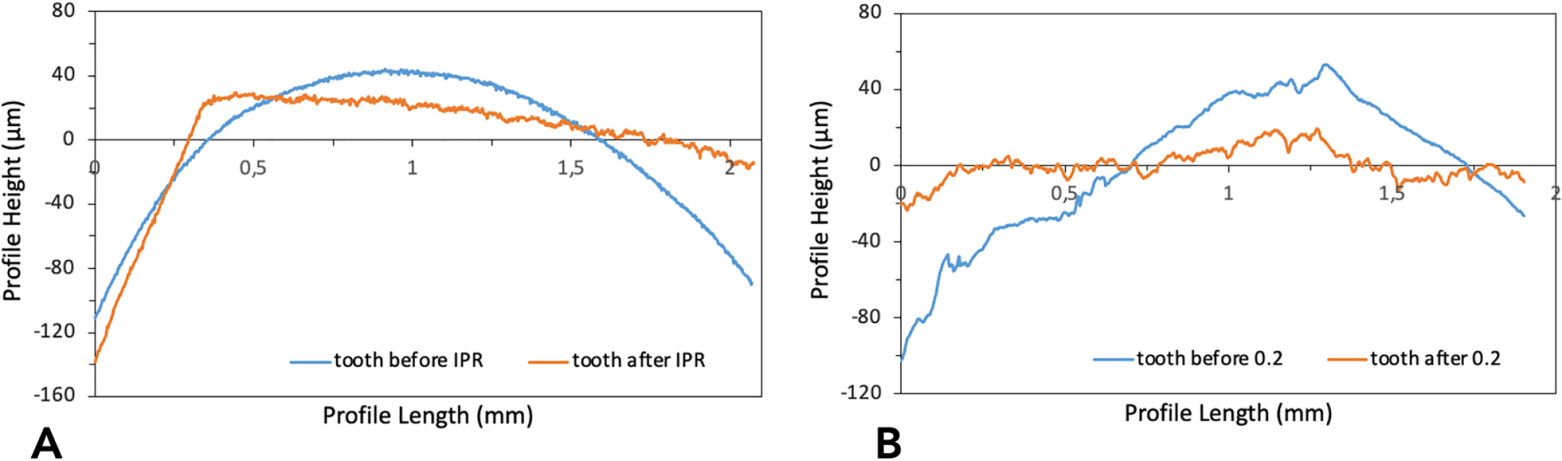
Roughness and waviness trend observed on enamel surfaces. **A** After complete oscillating IPR sequence (Group 1). **B** After single metallic oscillating strip (Group 2)

**Fig. 4:**
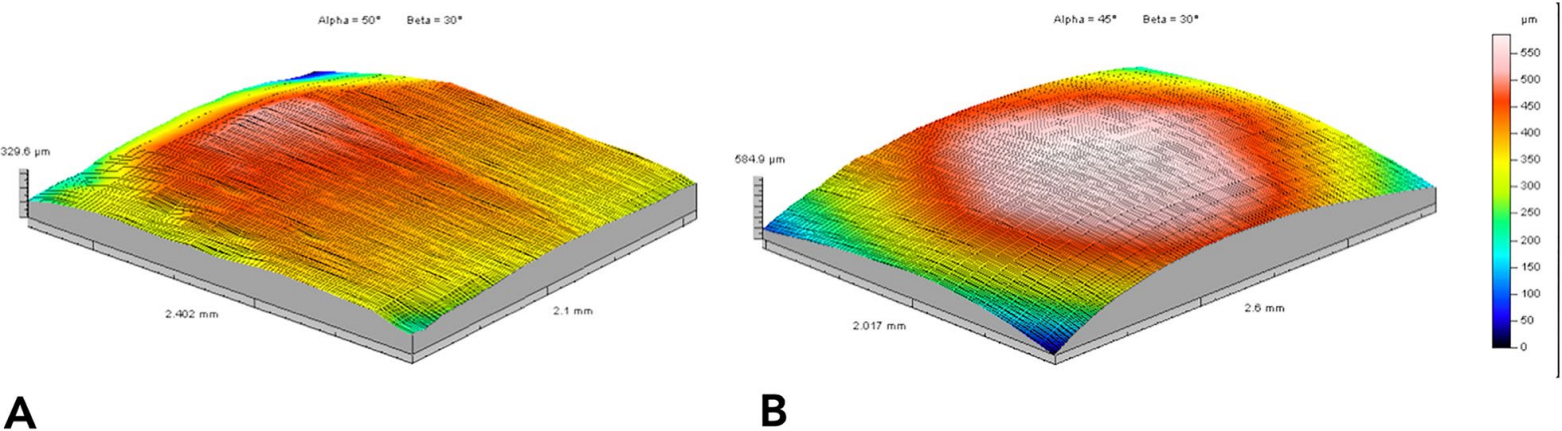
3D maps of the treated enamel surfaces. **A** Enamel profile after complete oscillating IPR sequence sliding test (Group 1). **B** Enamel profile after sliding test with single metallic strip of 0.2 mm (Group 2)

**Fig. 5:**
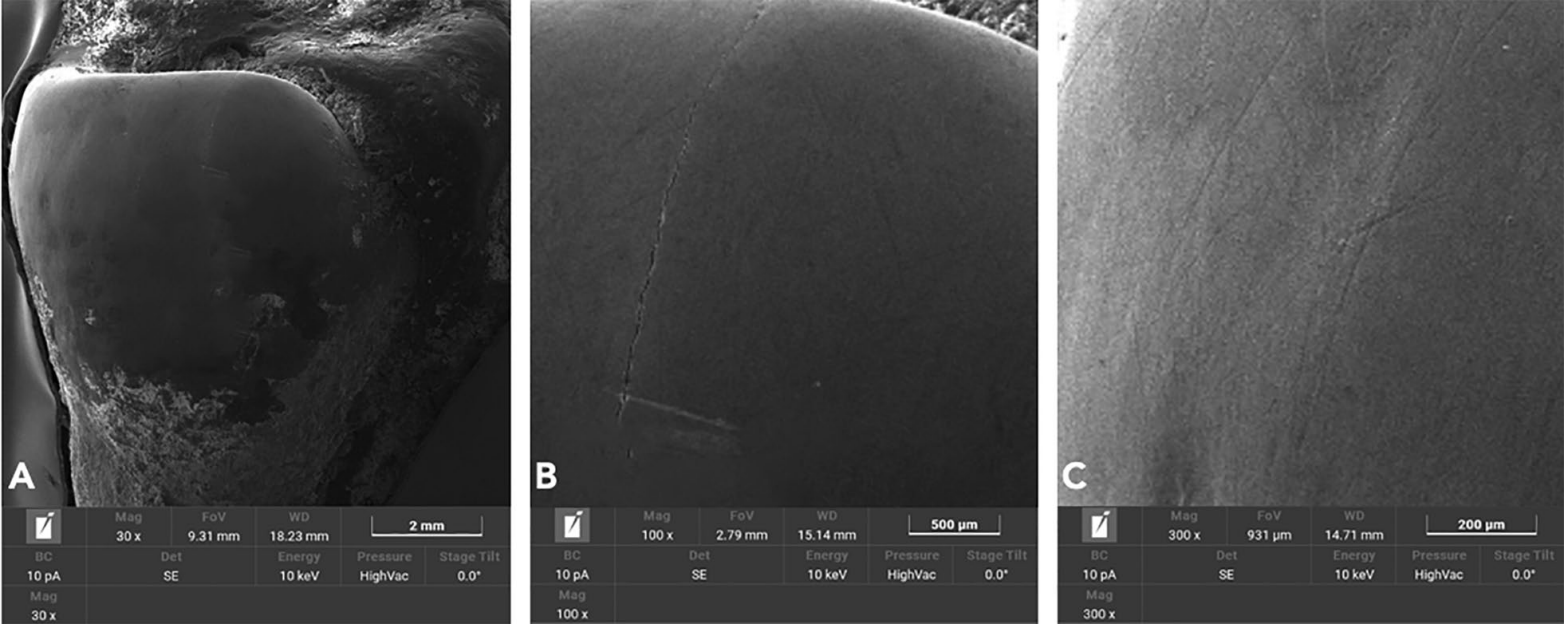
SEM analysis of untreated enamel surfaces underwent complete oscillating IPR sequence (Group 1). **A** 30X. **B** 100X. **C** 300X

**Fig. 6:**
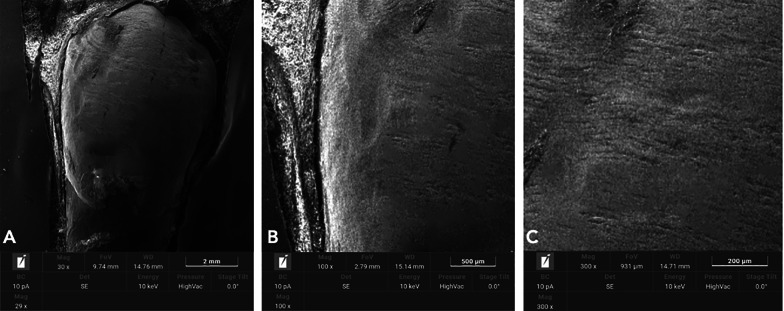
SEM analysis of untreated enamel surfaces underwent single metallic strip of 0.2 mm (Group 2). **A** 30X. **B** 100X. **C** 300X

**Fig. 7:**
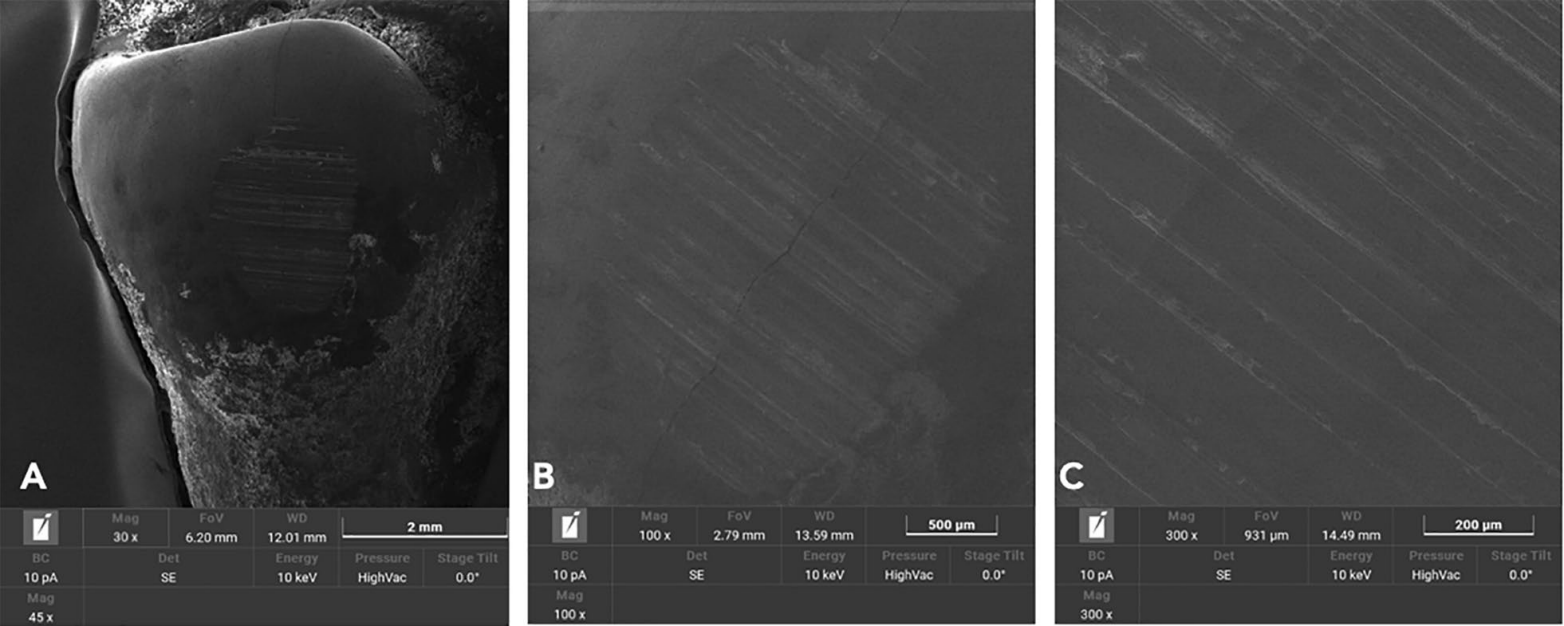
SEM analysis of enamel surfaces after complete oscillating IPR sequence (Group 1). The time sliding was set at 30 s for each strip. **A** 30X. **B** 100X. **C** 300X

**Fig. 8:**
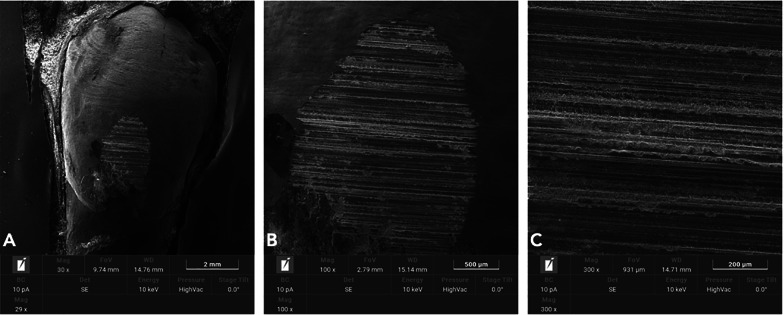
SEM analysis of enamel surfaces after single metallic strip of 0.2 mm (Group 2). The time sliding was set at 150 s. **A** 30X. **B** 100X. **C** 300X

Descriptive statistics and statistical comparisons of the surface roughness and waviness measurements obtained by contact probe surface profiler (TalySurf CLI 2000; Taylor Hobson, Leicester, UK) are summarized in Table 1. Significant differences were found between the tested samples in terms of surface roughness and waviness. Enamel surfaces after complete IPR sequence showed lower values of surface roughness (Ra − 0.34 µm, Rt − 1.55 µm) when compared with those treated with the single strip. As for profile waviness, a different trend was observed. Statistical comparison revealed a significant increase of waviness parameters (Wa 0.25 µm, Wt 4.02 µm) when teeth underwent complete IPR oscillating system. Different trends of roughness and waviness measurements are shown in Fig. 3. The 3D maps of worn surfaces for both groups are reported in Fig. 4. Although the extension of the surface involved is comparable among the two samples, the volume revealed a more deeply worn area after use of the single metallic oscillating strip. SEM evaluation (30X, 100 X and 300X) of enamel surface before and after the test is shown in Figs. 5, 6, 7, and 8. Enamel surface underwent Group 1 showed smoothers and more homogeneous profiles (Figs. 5 and 7) when compared with one treated with Group 2 (Figs. 6 and 8). Qualitatively evaluation carried out by SEM analysis clearly revealed different shapes and dimensions of the incisions produced by the complete IPR sequence and the metallic strip (Figs. 7 and 8). Oscillating IPR sequence defined more regular surfaces with some light parallel lines with some minor grooves of 1–3 μm and a more uniform enamel coating (Score 1). Oscillating metallic 0.2 mm strips revealed irregular surfaces characterized by extended grooves, alternated with enamel ridges and irregular fragment. This configuration corresponds to a Score 3 according to Nucci’s enamel surface classification.

**Table 1:** Descriptive statistics and statistical comparisons (independent samples *t*-test) of the surface roughness and waviness measurements between enamel undergone the complete IPR oscillating system (Group 1) and enamel treated with single oscillating metallic strip (Group 2)

Variables	Enamel after IPR sequence		Enamel after single strip		Comparison		95% CI of the difference	
	Mean	SD	Mean	SD	Diff.	*P* value	Lower	Upper
Ra (µm)	0.45	0.04	0.79	0.08	0.34	**0.000**	− 0.01	0.91
RSm (µm)	0.02	0.01	0.03	0.02	− 0.01	0.185	− 0.09	0.11
Rt (µm)	4.57	0.58	6.12	0.82	− 1.55	**0.000**	3.46	− 10.24
Wa (µm)	2.16	0.13	1.91	0.16	− 0.25	**0.000**	− 3.31	2.95
Wt (µm)	11.58	1.73	7.56	0.66	− 4.02	**0.000**	− 0.172	1.118

*Ra* arithmetic mean roughness value; *RSm* mean peak width; *Rt* total height of the roughness profile; *Wa* arithmetic mean waviness value; *Wt* total height of the waviness profile; *µm* micrometer; *SD* standard deviations; *Diff.* differences; *CI* confidence interval

## Discussion

Increasing demand of alternative procedures to extraction treatments promoted the introduction of several IPR systems [1, 4–7, 9, 10]. Most common are represented by manual abrasive strips, mechanical oscillating abrasive systems, diamond-coated segmented disks, and rotating diamond burs [1, 8–10, 15]. Recently, mechanical oscillating abrasive strips have gained in popularity [8, 10]. Some authors highlighted various advantages of this system in comparison with more traditional ones: avoiding risk of cutting into the soft tissue, possibility of more regular enamel surface, and more predictable results [11, 16–18]. Several studies [8, 10, 15] concluded that mechanical IPR systems reduce chairside time compared to manual strips. In contrast, manual abrasive strips are particularly indicated for anterior teeth, rotated elements, and recontouring procedures [1, 4]. However, they can result impractical, unproductive, and time-consuming when used for posterior teeth [1, 4–10, 15]. Many studies [8, 11, 12, 16, 19–22] assessed the clinical effects that IPR can have on the enamel surface [8, 11, 12, 16, 19–22]. In terms of superficial modifications, Bonetti et al. and Arman et al. [16, 19] concluded that all stripping methods significantly roughened the enamel surfaces. Baumgartner et al. [22] concluded that grinding with mechanical oscillating systems resulted in rougher enamel surfaces in comparison to untreated ones. However, no studies in literature analyzed the effects of a complete clinical IPR sequence on biological structures. In the present investigation, enamel surface appeared rougher than the untreated control after both IPR procedures. Mechanical IPR protocol (Group 1) produced a more regular enamel surface in comparison with the metallic oscillating strip of 0.2 mm (Group 2). The IPR protocol [9] including opening phase, interproximal enamel removal, and finishing and polishing phases should preserve the enamel integrity and morphology after interproximal enamel reduction process (Fig. 5). From a clinical point of view, the respect of the operative sequence allows a gradual access to the interproximal surfaces avoiding overpressure at the level of both periodontal and dental structures. The increasing abrasive capacity of the strips removes gradual amounts of enamel leaving minimum and more regular residual roughness on the treated surfaces. On the other hands, the strips dedicated to the finishing and polishing phases are fundamental for a further smoothing of the enamel as it can be noticed by the results observed in this study. According to our results, Kaaouara et al. [8, 23] revealed that mechanical oscillating diamond strips system produced more regular surface, with light parallel lines and minor grooves than manual abrasive strips. The study highlighted the importance of the finishing step to reduce the profile abrasions defined by IPR leaving surface conditions more similar to untreated enamel surfaces. The experimental analysis of the metallic strips of 0.2 mm revealed more irregular surface with extended groves, enamel ridges, and irregular fragments (Fig. 8), suggesting that the single use of a metallic abrasive strips could be considered efficient in terms of enamel reduction but not respectful of biological structures. The use of the only abrasive metallic strips implies the immediate removal of the enamel with a consequent increase of the residual irregularities. A possible polishing phase performed on these highly worn surfaces would leave them smoother but still irregular and with grooves. The quantitative comparison of surface roughness and waviness of enamel surfaces (Table 1, Fig. 3) revealed more regular areas when the complete IPR oscillating sequence was applied. Surface roughness values showed decreased trend after the sequence and more homogeneous surfaces without scratches and irregularities in agreement with the qualitative evaluation (Fig. 7). On the other hand, a significant increase of Wa and Wt parameters was observed revealing more stable and regular macroscopic morphology after the sequential use IPR system strips. Considering the existing literature [8, 11, 14–16, 24, 25] and the findings obtained on the necessity of an adequate polishing after IPR to guarantee a good long-term prognosis, enamel surfaces should be polished after all IPR procedures. Moreover, the clinical execution of IPR in compliance with the protocol increases the accuracy of the technique allowing the achievement of the enamel reduction amount required by the treatment. A limitation of the present study design was the likelihood of spurious inferences that could affect the results, such as the access to the interproximal point, the severity of crowding, variability in tooth morphology, and the bias related to operator ability.

## Conclusions

Qualitative SEM analysis showed a more regular enamel surfaces when teeth undergone the mechanical IPR protocol with respect to the use of a single metallic strip. The clinical standardization of an IPR clinical sequence increases the accuracy of enamel reduction required by the treatment and helps the clinician to adopt this non-extractive treatment procedure in the respect of the biological structures. An adequate polishing after IPR plays a crucial role to guarantee a good long-term prognosis and enamel morphologic integrity.

## Data Availability

All data generated or analyzed during this study are included in this published article.

## Abbreviations

IPR: Interproximal reduction
SEM: Scanning electronic microscope
3D: Three dimensional
Ra: Arithmetic mean roughness value
RSm: Mean peak width
Rt: Total height of the roughness profile
Wa: Arithmetic mean waviness value
Wt: Total height of the waviness profile
µm: Micrometer
